# Wide Spread of *bla*_CTX–M–9_/*mcr-9* IncHI2/ST1 Plasmids and CTX-M-9-Producing *Escherichia coli* and *Enterobacter cloacae* in Rescued Wild Animals

**DOI:** 10.3389/fmicb.2020.601317

**Published:** 2020-11-17

**Authors:** Marisa Haenni, Véronique Métayer, Romane Jarry, Antoine Drapeau, Marie-Pierre Puech, Jean-Yves Madec, Nicolas Keck

**Affiliations:** ^1^ANSES, Laboratoire de Lyon, Unité Antibiorésistance et Virulence Bactériennes – Université de Lyon, Lyon, France; ^2^Laboratoire Départemental Vétérinaire de l’Hérault, Montpellier, France; ^3^Hôpital pour la Faune Sauvage Garrigues-Cévennes, Ganges, France

**Keywords:** CTX-M, *mcr-9*, wild bird, *E. coli*, *E. cloacae*, IncHI2

## Abstract

Wildlife has recently been pinpointed as one of the drivers of dissemination of genes conferring resistances to clinically important antimicrobials. The presence of both extended-spectrum beta-lactamase- (ESBL) and carbapenemase-encoding genes has notably been reported in wild birds, that can act as sentinels of antimicrobial resistance (AMR) contamination but also as long-distance spreaders in case of migratory birds. Here, 424 wild birds brought to a rescue center in France were sampled over a 6-month period. These birds encompassed 62 different sedentary or migratory species. A further 16 wild mammals present in the center were also investigated. No carbapenemase-producer was found, but we identified a surprisingly high proportion (24.1%) of ESBL-positive isolates. A total of 144 non-duplicate isolates were collected, including *Escherichia coli* (*n* = 88), *Enterobacter cloacae* (*n* = 51), and *Citrobacter freundii* (*n* = 5), of which 123 carried the *bla*_CTX–M–__9_ gene. PFGE, phylogroup, and MLST revealed the presence of a limited number of ESBL-positive clones circulating in these animals, all presenting multiple associated resistances. Next-generation sequencing on a subset of isolates, followed by Southern blot hybridization, showed the wide dissemination of an IncHI2/ST1 plasmid carrying the *bla*_CTX–M–__9_, *bla*_SHV–__12_ and *mcr-9* genes. In all, our results undoubtedly reflect cross transmissions of ESC-resistance (ESC-R) Enterobacteriaceae within the rescue center – similarly to nosocomial spreads observed at hospital, rather than the true bacterial flora of birds. We also showed that the spread of ESC-R in this rescue center did not only rely on clonal but also on a highly successful plasmidic transmission. Since most animals are intended to get back to nature after a few days or weeks, this is obviously an issue with regard to ESBL dissemination in natural environments.

## Introduction

The importance of wildlife in disseminating genes conferring resistances to clinically important antimicrobials (CIAs), such as to extended-spectrum cephalosporins (ESC) or carbapenems (CP), has been recurrently highlighted (Wang et al., 2017; Dolejska and Literak, 2019). Antimicrobial resistance (AMR) in wildlife most likely reflects the pollution of natural environments with AMR genes, plasmids or bacterial clones selected in non-wildlife sectors. AMR acquisition most probably occurs through food opportunities – including dejections from domestic animals or polluted lands – and water sources, but the exact origin of AMR in specific wild individuals is usually unknown (Guenther et al., 2011; Mukerji et al., 2020). In most cases, AMR in wildlife has been found incidentally and, to date, most publications refer to wild birds, which encompass a wide diversity of animal species, habits and behaviors.

In wild birds, major *Escherichia coli* clones circulating in humans were detected, such as of sequence type (ST)131, ST410, ST648, or ST38 to only name a few, suggesting that these animals were indirectly contaminated by human sources (Schaufler et al., 2016; Atterby et al., 2017; Guenther et al., 2017; Yang et al., 2019). In rare occasions, AMR epidemiology in wild birds more clearly mirrored the one in humans and/or domestic animals in the same country, thereby supporting the hypothesis of local cross-sectorial transmissions. It was notably exemplified for CTX-M-producing Enterobacteriaceae in Sweden and Canada (Bonnedahl et al., 2015; Atterby et al., 2017) or IMP-4-producing Enterobacteriaceae in Australia (Dolejska et al., 2016). In addition to being sentinels of AMR contamination from other sectors, migratory birds can also spread AMR genes or antimicrobial resistant bacteria over very long distances, whose impacts on public health are suspected but not fully clarified (Guenther et al., 2012; Fuentes-Castillo et al., 2019).

At a global scale, CP- or colistin resistance in wild birds has been much less reported than ESC-resistance (ESC-R). It may however reflect a lack of studies, or the accumulation of case reports that do not accurately reflect the true epidemiology. Indeed, investigations on AMR in wild birds sometimes revealed unexpected reservoirs, such as recently where a high proportion of CP-resistant NDM-5-producing *Klebsiella pneumoniae* was observed in migratory birds in China (Liao et al., 2019). With regard to colistin resistance, a limited number of studies reported the plasmid-mediated *mcr-1* gene in wild birds (Tarabai et al., 2019). In France, ESBL-producing Enterobacteriaceae were detected in 2009 in 17/90 (18.9%) of juvenile yellow-legged gulls (Bonnedahl et al., 2009), while the sporadic detection of a VIM-1-producing *E. coli* was reported in 2012 in the same area (South of France) and the same bird species (Vittecoq et al., 2017). Recently, multi-drug resistant Enterobacteriaceae were again found in yellow-legged gulls in Marseille, France (Ngaiganam et al., 2019).

In all, several works investigating AMR in wild birds resulted from convenient samples from dead animals. Numerous studies were also based on fecal dejections collected in various places where birds live or transit, such as landfills, beaches, urban parks, nests, and other habitats, but associating fecal samples to the right bird species may be challenging. In some situations however, these approaches valuably allowed studying AMR in large cohorts of individuals of the same bird species in their natural environment, as for instance illustrated for black kites (*Milvus milvus*), corvids (*Corvus brachyrhynchos*, *Corvus corax*), gulls (*Larus glaucescens, Larus ridibundus*), pigeons (*Columba livia*) or storks (*Ciconia ciconia*) (Bonnedahl et al., 2010, 2014; Jamborova et al., 2018; Tarabai et al., 2019; Zendri et al., 2020). Here, we adopted a different strategy by systematically sampling all incoming wild birds at a French rescue center over a 6-month period. Such a systematic sampling design, which can for example be set up in rescue centers or during ringing campaigns (Guenther et al., 2010; Schaufler et al., 2016), is still rare in the field of AMR in wild birds. Both sedentary and migratory species were considered and sampled, and AMR genes, plasmids and bacterial clones were further investigated at phenotypic and molecular levels. Wild mammals present in the center were also studied. Our data support interesting hypotheses on the spread of AMR at the interface of wild animals and human communities.

## Materials and Methods

### Bacterial Isolates

Between April and November 2015, 424 migratory and sedentary birds were sampled at an animal rescue center in the Hérault department, South of France. Cloacal sample was taken using Eswab minitip or pernasal flocked (Labelians, Nemours, France), depending to the size of the bird. Sampling was performed in the first days after arrival at the center, ranging from 24 h to 10 days. During the study, and for epidemiological reasons (see section “Results”), 16 mammals which were temporarily hosted in the rescue center were also sampled using the same procedure as for birds. Within 24 h after sampling, samples were plated on Drigalsky agar as a control of growth, as well as on the selective ChromID ESBL, ChromID OXA-48 and CarbaSMART media for the detection of ESC- and CP resistance. One colony per morphology was picked up and identified by MALDI TOF (VITEK MSVersion 3.0, bioMérieux, Marcy L’Etoile, France).

### Antimicrobial Susceptibility Testing

Susceptibility testing was performed by disc diffusion on Mueller-Hinton agar (Bio-Rad, Marne-la-Coquette, France), according to the guidelines and clinical breakpoints of the Antibiogram Committee of the French Society for Microbiology^1^. The following discs of human and/or veterinary interest were tested: amoxicillin, amoxicillin + clavulanic acid, cefalotin, cefuroxime, cefotaxime, ceftiofur, ceftazidime, cefoxitin, cefepime, aztreonam, cefquinome, ertapenem, streptomycin, kanamycin, amikacin, apramycin, gentamicin, tobramycin, netilmicin, chloramphenicol, florfenicol, tetracycline, colistin, sulfonamides, trimethoprim, nalidixic acid, and enrofloxacin. *E. coli* ATCC 25922 was used as a quality control. Minimum inhibitory concentration (MIC) for colistin was determined by the broth microdilution method, as recommended by EUCAST (EUCAST, 2016).

### Identification of β-Lactamase Genes

PCRs were performed using specific primers for the detection of *bla*_TEM_, *bla*_SHV_, *bla*_CTX–M_ group 1, group 2, and group 9 (Shibata et al., 2006; Dierikx et al., 2010). For all *bla*_CTX–M_ group 1 and group 9-positive isolates, additional PCRs were performed using the primers ISEcp1L1/P2D and MA1/MA2, respectively. The *bla*_CMY_ genes were detected using CF1/CF2 primers (Eckert et al., 2004). All positive amplicons were sequenced (Genewiz, London, United Kingdom). The *mcr-1* to *mcr-5* genes were detected using published primers (Lescat et al., 2018) while detection of the *mcr-9* gene was performed using the primers mcr9_int_for (5′-GAAACTAACCCCCAGGAAGC) and mcr9_int_rev (5′-TTTTGGCGATTTCATCATCA).

### Genetic and Molecular Typing of the Strains

Phylogenetic grouping of the *E. coli* isolates was performed using the improved method described by Clermont et al. (2013). PFGE was performed on *Xba*I-digested DNA. Multi-locus sequence typing (MLST) was performed on one representative of each PFGE profile according to the following websites: https://pubmlst.org/bigsdb?db=pubmlst_mlst_seqdef for *E. coli* according to the Achtman scheme, and https://pubmlst.org/ecloacae/ for *E. cloacae*.

### Whole Genome Sequencing

Genomic DNA of eight selected isolates (four *E. coli* and four *E. cloacae*) was extracted from an overnight culture using the GmbH & Co. KG – NucleoSpin^®^ Microbial DNA (Macherey Nagel, Germany). Whole genome sequencing was performed using the NovaSeq technology (Illumina). The paired-end reads (average read length of 151 bp) were generated with a 69-fold to 131-fold coverage. After Trimmomatic cleaning, *de novo* assembly was performed using Shovill (version 0.9.0). Resistance genes were searched from the assembled genomes using the ResFinder database (CGE^2^). Pairwise single nucleotide polymorphism (SNP) distances were calculated from core genome alignments generated by Roary using snp-dists^3^.

### Plasmid Characterization

Plasmids were typed by PCR-based replicon typing (PBRT) according to the PBRT kit scheme described by Carattoli et al. (2005) using a commercial kit (Diatheva, Cartoceto, Italy). PFGE-S1 gels were performed, followed by Southern blot using adequate probes (*bla*_CTX–M–__9_, *bla*_SHV–__12_, *mcr-9*, IncHI2) according to the manufacturer’s protocol (Roche Diagnostics, Meylan, Germany). Plasmid co-localization was assessed by comparison between the bands corresponding to the resistance genes and those corresponding to the Inc type of the plasmid.

### Ethics Statement

No ethical approval was needed since this study did not involve any experimentation on animals.

### Accession Number(s)

The whole genome shotgun project was deposited in DDBJ/EMBL/GenBank under the BioProject accession number PRJNA659767.

## Results

Over the 6-month period, a total of 424 wild birds were sampled, that belonged to 62 different species distributed into 25 sedentary ones and 37 species migrating either inside Europe or to Africa. All of these species were represented by less than 20 individuals, except for black swifts (*Apus apus*, *n* = 74), rock pigeon (*C. livia*, *n* = 55), Turkish turtledove (*Streptopelia decaocto*, *n* = 31), and black-billed magpie (*Pica pica*, *n* = 21). The vast majority of the birds were referred to the rescue center after serious injury (various traumas, such as broken legs or wings) or because they had been found incidentally. In some occasions, related juveniles from the same litter were collected at the same time. Altogether, all birds were confirmed to be devoid of obvious bacterial or viral infection after veterinary examination so that any AMR in the positive animals should be considered as carriage.

A total of 102 birds (102/424, 24.1%) were positive for the presence of at least one ESC-R Enterobacteriaceae, while no CP-resistant isolate was identified (Supplementary Table 1). Forty-six positive birds were sedentary (14 different species including ducks, sparrows and gulls) while 56 were migratory (19 different species either migrating in Europe, such as tawny owls or in Africa, such as swallows). No bird species was more represented amongst the positive individuals compared to the negative ones. Different bacterial morphologies were identified on numerous selective plates, but only non-duplicate isolates (based on the PFGE profile, phylogroup, CTX-M-type and the antibiogram) were kept for further analysis. Multiple ESC-R isolates were identified in 36/102 samples (*n* = 35.3%). For each positive animal, one to four different bacterial morphologies were identified, so that a total of 144 different isolates were collected (Supplementary Table 1). ESC-R Enterobacteriaceae were identified as *E. coli* (*n* = 88), *Enterobacter cloacae* (*n* = 51), and *Citrobacter freundii* (*n* = 5). One ESC-R *E. coli* and one ESC-R *E. cloacae* were concomitantly identified in 22/102 samples (*n* = 19.6%), and the same CTX-M-9 enzyme was found in concomitant *E. coli* and *E. cloacae* isolates in 20/22 samples.

The *bla*_CTX–M–__1_ gene was found in 21 isolates (20 *E. coli* and 1 *E. cloacae*), while the *bla*_CTX–M–__9_ gene was dominantly detected in the 123 remaining isolates, i.e., in 68 *E. coli*, 50 *E. cloacae* and five *C. freundii* isolates (Supplementary Table 1). All isolates presented multiple associated resistances (Table 1), the most frequent ones being to sulfonamides (100% in *E. cloacae*; 98.9% in *E. coli*), tetracyclines (78.4 and 85.2%, respectively) and chloramphenicol (90.2 and 72.7%, respectively). *E. cloacae* isolates were also often resistant to gentamicin (64.7%) and enrofloxacin (25.5%). No resistance to amikacin or colistin was detected.

**TABLE 1:** Phenotypic resistances associated to ESBL-producing *E. coli* and *E. cloacae*.

**Antibiotic**	***E. coli* (*n* = 88)**		***E. cloacae* (*n* = 51)**	
	**Number**	**Percentage**	**Number**	**Percentage**
Streptomycin	22	25.0	14	27.5
Kanamycin	61	69.3	40	78.4
Amikacin	0	0.0	0	0.0
Apramycin	0	0.0	0	0.0
Gentamicin	35	39.8	33	64.7
Tobramycin	61	69.3	45	88.2
Netilmicin	57	64.8	39	76.5
Chloramphenicol	64	72.7	46	90.2
Florfenicol	1	1.1	0	0.0
Tetracycline	75	85.2	40	78.4
Colistin*	0	0.0	0	0.0
Sulfonamides	87	98.9	51	100.0
Triméthoprim	19	21.6	13	25.5
Nalidixc acid	12	13.6	21	41.2
Enrofloxacin	5	5.7	13	25.5

*As determined using the micro-dilution method.

The vast majority of *E. coli* isolates belonged to the phylogroups A (*n* = 32) and B1 (*n* = 53) usually associated to commensal isolates, while only three belonged to the more virulent B2 (*n* = 2) and D (*n* = 1) phylogroups. A total of 22 PFGE profiles and 14 different STs were identified among the *E. coli* isolates (Supplementary Table 1 and Table 2, also see Supplementary Figures 1, 2). The two PFGE profiles A and B were dominant and represented 63.6% (56/88) of the *E. coli* isolates. These two PFGE profiles corresponded to ST746 (*n* = 30) and ST1246 (*n* = 27), respectively, and both produced CTX-M-9. ST155 (*n* = 14) was also recurrently found and produced CTX-M-1. This ST was more heterogeneous than ST746 and ST1246 since six different PFGE profiles were identified. Dynamics of these three main *E. coli* lineages over the 6-month period showed that ST1246 sporadically but regularly occurred between May and July, while ST746 had a more epidemic behavior, with 25 isolates detected between the end of June and mid-July (Supplementary Figure 3). For *E. cloacae* as well, most isolates distributed into a limited number of STs and PFGE profiles and also presented a peak of occurrence between the end of June and mid-July. Indeed, *E. cloacae* isolates mainly belonged to the new ST corresponding to a single locus variant of ST714 (*n* = 16; allelic sequence 2/2/gyrB^∗^/133/51/2/14), ST135 (*n* = 11), ST78 (*n* = 10), and ST104 (*n* = 9), representing 90% (46/51) of all isolates. Contrary to *E. coli*, no *E. cloacae* isolate produced CTX-M-1.

**TABLE 2:** Sequence types (ST) of ESBL-producing *E. coli* and *E. cloacae*.

**ST**	**ESBL enzyme**	**Phylogroup**	**Number of isolates**	**Number of PFGE profiles**
***E. coli***				
746	CTX-M-9	A	30	1
1246	CTX-M-9	B1	26	1
155	CTX-M-1	B1	14	6
40	CTX-M-9	B1	3	1
224	CTX-M-1	B1	3	3
223	CTX-M-9	B1	2	1
4054	CTX-M-9	B1	2	1
10	CTX-M-1	A	1	1
88	CTX-M-1	B1	1	1
136	CTX-M-1	B2	1	1
162	CTX-M-9	B1	1	1
174	CTX-M-9	D	1	1
1643	CTX-M-9	B2	1	1
ND^1^	CTX-M-9	A	1	1
ND	CTX-M-9	A	1	1
***E. cloacae***				
New (SLV ST714)	CTX-M-9	–	16	1
135	CTX-M-9	–	11	1
78	CTX-M-9	–	10	1
104	CTX-M-9	–	9	1
ND	CTX-M-9	–	1	1
ND	CTX-M-9	–	1	1
ND	CTX-M-9	–	1	1
ND	CTX-M-9	–	1	1
ND	CTX-M-1	–	1	1

*^1^ND, not done.*

During the 6-month sampling period, a few wild mammals (16) were also healed in the rescue center, using the same facilities as for birds, and that were sampled and analyzed using the same procedures as for birds. Bacterial identification and antimicrobial susceptibility testing proved that eight of these animals carried ESBL-producing *E. coli* isolates (Table 3 and Supplementary Table 1), including one rabbit (*Oryctolagus cuniculus*), two hares (*Lepus europaeus*), one squirrel (*Sciurus vulgaris*), and four foxes (*Vulpes vulpes*). Further molecular analysis of ESBL genes together with PFGE and MLST determination concluded that the rabbit, the two hares and one fox carried an ST223 CTX-M-9-producing *E. coli*. The three other foxes carried an ST155 CTX-M-1-producing *E. coli* while the squirrel carried an ST1246 CTX-M-9-producing *E. coli*. All these CTX-M-producing *E. coli* lineages and corresponding PFGE profiles had also been detected in birds. No *E. cloacae* was detected in wild mammals.

**TABLE 3:** Characteristics of the eight *E. coli* isolates collected from mammals in the rescue center.

**Animal number**	**Strain number**	**Sampling date**	**Animal species**	**Phylogeny**	**PFGE profile**	**MLST**	**CTX-M enzyme**
A1_140	40430	15/06/2015	*Oryctolagus cuniculus*	B1	C	ST223	CTX-M-9
A2_143	40431	16/06/2015	*Lepus europaeus*	B1	C	ST223	CTX-M-9
A3_144	40432	16/06/2015	*Lepus europaeus*	B1	C	ST223	CTX-M-9
A4_145	40433	16/06/2015	*Sciurus vulgaris*	B1	B	ST1246	CTX-M-9
A5_166	40440	18/06/2015	*Vulpes vulpes*	B1	C	ST223	CTX-M-9
A6_167	40441	18/06/2015	*Vulpes vulpes*	B1	O	ST155	CTX-M-1
A7_168	40442	18/06/2015	*Vulpes vulpes*	B1	O	ST155	CTX-M-1
A8_169	40443	18/06/2015	*Vulpes vulpes*	B1	O	ST155	CTX-M-1

To further characterize the isolates circulating in the rescue center, four couples of CTX-M-9-producing *E. coli*/*E. cloacae* (each couple originating from a single bird) were fully sequenced using Illumina technologies (Table 4). The four bird species were a magpie (*P. pica*), a jackdaw (*Corvus modenula*), an eagle owl (*Bubo bubo*), and a crag martin (*Ptyonoprogne rupestris*). Except *P. rupestris* which has a long-distance migratory behavior (Africa), the three other species are considered sedentary. The four *E. coli* isolates were from the two main lineages (ST746 and ST1246) found in this study, with the *B. bubo* and *P. rupestris* harboring a ST746 *E. coli*, and the *P. pica* and *Corvus modenula* harboring a ST1246 *E. coli*. SNP analysis on the core genome proved that isolates from a same ST were genetically highly similar, which differed by respectively, 8 and 21 SNPs. The same genomic similarities were found among the two *E. cloacae* of the same ST in the corresponding birds; which differed by respectively 29 and 63 SNPs.

**TABLE 4:** Epidemiological and molecular features of the eight isolates that were fully sequenced.

**Strain**	**Common name (species)**	**Migratory behavior**	**Sampling date**	**Bacterial species**	**MLST**	**ESBL genes**	**CTX-M-carrying plasmid**	**Resistance genes**
40412	Magpie (*Pica pica*)	Sedentary	03/06/2015	*E. coli*	1246	*bla*_CTX–M–__9_, *bla*_SHV–__12_	IncHI2/ST1	*aac*(6′)-*Ib3*, *aadA2b*, *ant*(2″)-*Ia*, *catA1*, *tet*(A), *sul1*, *mcr-9*, *aac*(6′)-*Ib-cr*, *qnrA1*
40435	Jackdaw (*Corvus modenula*)	Sedentary	16/06/2015	*E. coli*	1246	*bla*_CTX–M–__9_, *bla*_SHV–__12_	IncHI2/ST1	*aac*(6′)-*Ib3*, *aadA2b*, *ant*(2″)-*Ia*, *catA1*, *tet*(A), *sul1*, *mcr-9*, *aac*(6′)-*Ib-cr*, *qnrA1*
40460	Eagle owl (*Bubo bubo*)	Sedentary	30/06/2015	*E. coli*	746	*bla*_CTX–M–__9_, *bla*_SHV–__12_	IncHI2/ST1	*aac*(6′)-*Ib3*, *aadA2b*, *ant*(2″)-*Ia*, *catA1*, *tet*(A), *sul1*, *mcr-9*, *aac*(6′)-*Ib-cr*, *qnrA1*
40466	Crag martin (*Ptyonoprogne rupestris*)	Long distance (Africa)	30/06/2015	*E. coli*	746	*bla*_CTX–M–__9_, *bla*_SHV–__12_	IncHI2/ST1	*aac*(6′)-*Ib3*, *aadA2b*, *ant*(2″)-*Ia*, *catA1*, *tet*(A), *sul1*, *mcr-9*, *aac*(6′)-*Ib-cr*, *qnrA1*
40508	Magpie (*Pica pica*)	Sedentary	03/06/2015	*E. cloacae*	New*	*bla*_CTX–M–__9_, *bla*_SHV–__12_	IncHI2/ST1	*aac*(6′)-*Ib3*, *aadA2b*, *ant*(2″)-*Ia*, *catA1*, *tet*(A), *sul1*, *mcr-9*, *aac*(6′)-*Ib-cr*, *qnrA1*
40513	Jackdaw (*Corvus modenula*)	Sedentary	16/06/2015	*E. cloacae*	New	*bla*_CTX–M–__9_, *bla*_SHV–__12_	IncHI2/ST1	*aac*(6′)-*Ib3*, *aadA2b*, *ant*(2″)-*Ia*, *catA1*, *tet*(A), *sul1*, *mcr-9*, *aac*(6′)-*Ib-cr*, *qnrA1*
40522	Eagle owl (*Bubo bubo*)	Sedentary	30/06/2015	*E. cloacae*	78	*bla*_CTX–M–__9_	IncHI2/ST1	*aadA2b*, *ant*(2″)-*Ia*, *sul1*, *mcr-9*, *qnrS1*, *dfrA15*
40527	Crag martin (*Ptyonoprogne rupestris*)	Long distance (Africa)	30/06/2015	*E. cloacae*	78	*bla*_CTX–M–__9_	IncHI2/ST1	*aadA2b*, *ant*(2″)-*Ia*, *sul1*, *mcr-9*, *qnrS1*, *dfrA15*

*New variant which is a SLV of ST712 (allelic profile: 2/2/gyrB*/133/51/2/14).

NGS data also revealed additional information on the gene content, which was coherent with the resistance phenotypes observed (Table 4). Moreover, in all eight CTX-M-9-producing *E. coli* and *E. cloacae* of the four birds, NGS demonstrated the presence of the IncHI2/ST1 plasmid replicon and the *mcr-9* gene. Southern blot experiments on these eight isolates confirmed that *bla*_CTX–M–__9_ and *mcr-9* co-localized on the IncHI2/ST1 plasmid, together with the *bla*_SHV–__12_ gene when present. The presence of the *mcr-9* and *bla*_SHV–__12_ genes was further assessed by PCR, revealing that *bla*_SHV–__12_ was present in 99 and *mcr-9* in 118 out of the 123 CTX-M-9-producing isolates. Southern blot experiments performed on a representative sub-set of 21 CTX-M-9/SHV-12/MCR-9 positive *E. coli* and *E. cloacae* proved that these three genes systematically co-localized on an IncHI2/ST1 plasmid.

## Discussion

In this study, we report a high prevalence of 24.1% of ESC-R in a large collection of 424 wild birds sampled on arrival in an animal rescue center in France over a 6-month period. However, this high prevalence, moreover in a collection encompassing over 50 different bird species, must be cautiously interpreted. Indeed, when considering the delay between entrance and sampling (from 24 h to 10 days) together with the molecular evidence of a limited number of highly prevalent ESC-R clones colonizing the positive animals, this picture most likely reflects cross transmissions of ESC-R within the rescue center, similarly to nosocomial spreads observed at hospital. This interpretation is further reinforced by the evidence of ESBL carriage in several mammals kept in this center with the same bacterial lineages as observed in birds. The presence of ESBL-producing Enterobacteriaceae in wild mammals strongly suggests that they can potentially act as environmental vectors of resistance genes and/or resistant bacteria. This also alerts on the importance of separating animals (here mammals and birds) that can share AMR determinants before being released in nature. In all, the present situation does obviously not reflect the true prevalence of ESC-R in birds in the wild, but reveals a particular bacterial flora specific to the rescue center. A plausible hypothesis would be that some birds have been collected as naturally positive ESBL carriers, and that bird-adapted bacterial clones then colonized the environment of the rescue center – including aqueous solutions, sinks, cages or aviaries as shown in hospitals – thus favoring their persistence and dissemination (Lowe et al., 2012; Chapuis et al., 2016). In particular, *E. coli* ST746 had already been reported in birds in France in 2009, possibly suggesting specific adaptive properties (Bonnedahl et al., 2009). ST155 has also been reported in wild birds in many occasions (Hernandez et al., 2013; Alcalá et al., 2016).

The high prevalence of ESC-R *E. cloacae* isolates, often in co-occurrence with ESC-R *E. coli*, was another interesting feature of this study. Even though *E. cloacae* alone or as co-contaminant has been sporadically reported in birds (Giacopello et al., 2016), such a high prevalence was an unprecedented situation. It also most probably results from the intra-center spread of a limited number of ESC-R *E. cloacae* lineages, but this suggests that these lineages may be particularly adapted to the avian hosts. While ST104 and ST135 have only been sporadically reported, ST78 is also considered as a high-risk clone for humans and is a major driver of CP-resistance spread, notably in North America (Izdebski et al., 2015; Annavajhala et al., 2019). Whether this clone had a human source in our study or is also adapted to birds cannot be inferred from our results, and investigations on the microbiota of birds are clearly needed to better understand transmission routes of multi-drug resistant bacteria.

Interestingly, the spread of ESC-R in this rescue center did not only rely on clonal but also on plasmidic transmission. Here, we evidenced the wide dissemination of a single IncHI2/ST1 plasmid bearing *mcr-9*, *bla*_CTX–M–__9_, and in most cases *bla*_SHV–__12_. This plasmid was equally found in ESC-R *E. coli* and ESC-R *E. cloacae*, strongly suggesting that plasmid spread within the same bird has occurred as well. Of note, all isolates were phenotypically susceptible to colistin, which is a known feature of *mcr-9*, a gene that was first described in 2019 in *Salmonella enterica* from a human patient in the United States and since then has not been shown to confer phenotypic resistance to colistin (Carroll et al., 2019). The study by Chavda et al. (2019) strongly suggested that *mcr-9* was associated with large InHI2/IncHI2A plasmid, which was then corroborated by two other publications reporting *mcr-9*/*bla*_CTX–M–__9_/*bla*_VIM–__4_ in *E. cloacae* from a young patient in the United States and *mcr-9*/*bla*_SHV–__12_ in several Enterobacteriaceae from horses in Sweden on IncHI2 plasmids (Borjesson et al., 2019). The occurrence of *mcr-9* in both horses and birds may argue for an environmental dissemination and for a large epidemic success of these IncHI2/ST1 plasmids, thanks to plasticity and optimal conjugation properties at low temperatures (around 25°C) (Garcia-Fernandez and Carattoli, 2010). However, since *mcr-9* does not confer phenotypic resistance to colistin, its real role remains to be studied.

In terms of CTX-M epidemiology, the over-dominance of *bla*_CTX–M–__9_ in ESBL-positive animals was unexpected since *bla*_CTX–M–__9_ is very rare in France, both in domestic animals and humans (Robin et al., 2017). The *bla*_SHV–__12_ gene was also detected in a significant proportion of isolates, a gene that also remains infrequent in the current ESBL epidemiology in animals, except in some food-producing birds (broilers) in Europe. Therefore, one could argue that the environmental IncHI2 plasmid carrying *bla*_CTX–M–__9_, *mcr-9* and *bla*_SHV–__12_ may have been introduced occasionally by wild animals before displaying an epidemiological success in the rescue center. It is therefore to consider that veterinarians and all people in contact were highly exposed to ESC-R in this setting, which constitutes an open door for further ESBL spread in the community. Moreover, most animals in the rescue center are also intended to get back to nature after a few days or weeks, and this is obviously another issue with regard to ESBL dissemination in natural environments.

## Conclusion

In conclusion, to our best knowledge, this study highlights for the first time the broad dissemination of both ESC-R plasmid and ESC-R *E. coli* and *E. cloacae* clones in a rescue center for wild animals, mainly birds. It also reveals a probable dynamic transmission of ESBL genes between *E. coli* and *E. cloacae*, which can then further disseminate to the environmental settings, but also ultimately to people in contact and to wildlife once birds are released in nature. The exact source of ESC-R in this center is not easy to clarify. The nature of the ESBL genes found, which are rather rare in domestic animals and humans, may argue for an external reservoir but this remains speculative. Importantly however, this study highlights to what extent such settings at the interface between wildlife and non-wildlife sectors may act as critical points in the amplification of ESC-R prevalence. Even though the epidemic success of the IncHI2 plasmid carrying *bla*_CTX–M–__9_, *mcr-9* and *bla*_SHV–__12_ can be highlighted, the causes of such a wide dissemination of ESBL-producing bacteria inside the rescue center also remain unknown and may warrant investigation. Antibiotherapy was not common practice in this center (antibiotics were only prescribed in case of open fracture; M-PP, personal communication) but, as demonstrated in a recent study, a single source, such as the use of contaminated disinfectants, may be sufficient for a large and long-term contamination by resistant bacteria (Keck et al., 2020). Other hypotheses include frequent handling of animals, different birds kept in the same cage, difficulties to disinfect surfaces (often in wood) or inadequate hygiene procedures in often crowded settings, such as animal rescue centers. Since that study, the rescue center has implemented measures and procedures to avoid cross contaminations and control intra-center infections.

## Data Availability Statement

The datasets presented in this study can be found in online repositories. The names of the repository/repositories and accession number(s) can be found below: https://www.ncbi.nlm.nih.gov/genbank/, PRJNA659767.

## Ethics Statement

Ethical review and approval was not required for the animal study because no ethical approval was needed since this study did not involve any experimentation on animals. Samples used in this study were routine samples taken at the entry of wild birds in the recue center.

## Author Contributions

NK, MH, and J-YM designed the experiments, analyzed the data, and drafted the manuscript. M-PP performed the sampling campaign. RJ and VM performed the experiments. AD performed the NGS analyses. All authors approved the final version of this manuscript.

## Conflict of Interest

The authors declare that the research was conducted in the absence of any commercial or financial relationships that could be construed as a potential conflict of interest.

## Footnotes

1 https://www.sfm-microbiologie.org/

2 http://www.genomicepidemiology.org/

3 https://github.com/tseemann/snp-dists
