# Supplementary material for: Wide Spread of blaCTX–M–9/mcr-9 IncHI2/ST1 Plasmids and CTX-M-9-Producing Escherichia coli and Enterobacter cloacae in Rescued Wild Animals
Source: Front Microbiol. 2020 Nov 17;11:601317. doi: 10.3389/fmicb.2020.601317 (PMC7717979; doi:10.3389/fmicb.2020.601317)
Supplement: Supplementary file 1 [file Data_Sheet_1.PDF]

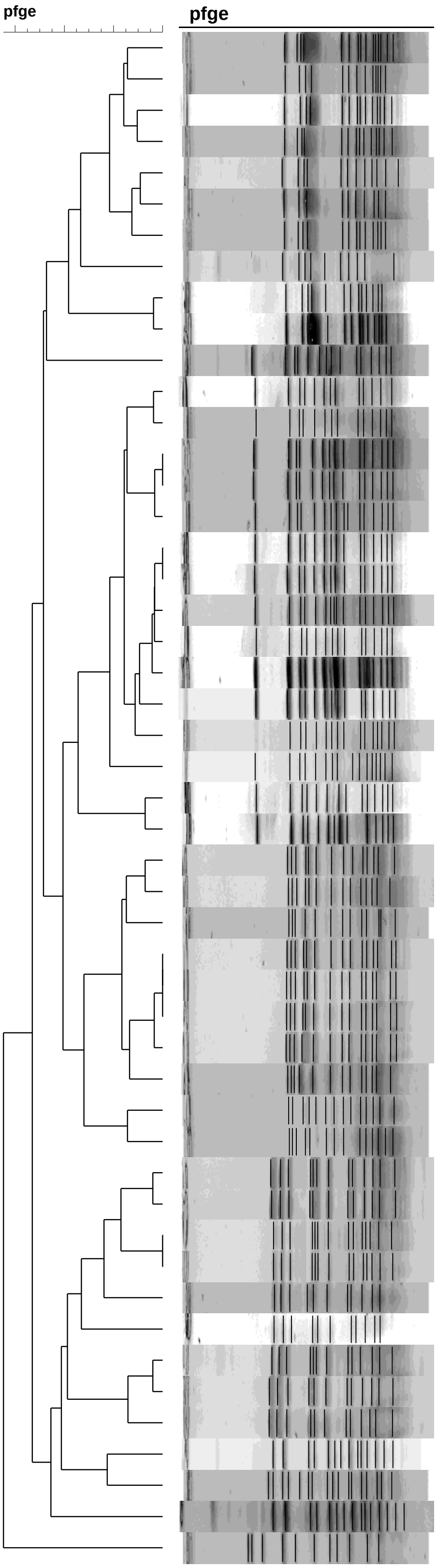


| Key | Bird number | Bacterial species | MLST | CTX-M enzyme |
|---|---|---|---|---|
| 40544 | 76_276_2 | E. cloacae | ST135 | CTX-M-9 |
| 41373 | 96_374 | E. cloacae | ST135 | CTX-M-9 |
| 40511 | 18_113 | E. cloacae | ST135 | CTX-M-9 |
| 40545 | 78_286 | E. cloacae | ST135 | CTX-M-9 |
| 40540 | 68_257_3 | E. cloacae | ST135 | CTX-M-9 |
| 41377 | 99_409 | E. cloacae | ST135 | CTX-M-9 |
| 40550 | 92_332 | E. cloacae | ST135 | CTX-M-9 |
| 40534 | 60_240_2 | E. cloacae | ST135 | CTX-M-9 |
| 40515 | 29_162_2 | E. cloacae | ST135 | CTX-M-9 |
| 40518 | 36_183_2 | E. cloacae | ST135 | CTX-M-9 |
| 40546 | 82_302_2 | E. cloacae | new | CTX-M-9 |
| 40508 | 11_93_2 | E. cloacae | new | CTX-M-9 |
| 41375 | 98_393 | E. cloacae | new | CTX-M-9 |
| 40542 | 71_267 | E. cloacae | new | CTX-M-9 |
| 40543 | 72_268_2 | E. cloacae | new | CTX-M-9 |
| 40547 | 88_313_2 | E. cloacae | new | CTX-M-9 |
| 40512 | 19_114 | E. cloacae | new | CTX-M-9 |
| 40513 | 27_15_2 | E. cloacae | new | CTX-M-9 |
| 40523 | 47_213 | E. cloacae | new | CTX-M-9 |
| 40514 | 28_157_2 | E. cloacae | new | CTX-M-9 |
| 40509 | 14_97_2 | E. cloacae | new | CTX-M-9 |
| 40408 | 7_66 | E. cloacae | new | CTX-M-9 |
| 40535 | 62_244 | E. cloacae | new | CTX-M-9 |
| 41369 | 94_347_2 | E. cloacae | new | CTX-M-9 |
| 40517 | 32_175_2 | E. cloacae | new | CTX-M-9 |
| 40519 | 38_186_2 | E. cloacae | new | CTX-M-9 |
| 40522 | 45_209_2 | E. cloacae | ST78 | CTX-M-9 |
| 40526 | 51_221_2 | E. cloacae | ST78 | CTX-M-9 |
| 40548 | 91_325 | E. cloacae | ST78 | CTX-M-9 |
| 40527 | 52_222_3 | E. cloacae | ST78 | CTX-M-9 |
| 40533 | 59_235 | E. cloacae | ST78 | CTX-M-9 |
| 40538 | 67_255 | E. cloacae | ST78 | CTX-M-9 |
| 40530 | 54_22_2 | E. cloacae | ST78 | CTX-M-9 |
| 40541 | 68_257_4 | E. cloacae | ST78 | CTX-M-9 |
| 41368 | 94_347 | E. cloacae | ST78 | CTX-M-9 |
| 41376 | 98_393_2 | E. cloacae | ST78 | CTX-M-9 |
| 40524 | 49_219 | E. cloacae | ST104 | CTX-M-9 |
| 40525 | 51_221 | E. cloacae | ST104 | CTX-M-9 |
| 40528 | 53_225_2 | E. cloacae | ST104 | CTX-M-9 |
| 40529 | 54_228 | E. cloacae | ST104 | CTX-M-9 |
| 40549 | 91_325_2 | E. cloacae | ST104 | CTX-M-9 |
| 40521 | 41_195_3 | E. cloacae | ST104 | CTX-M-9 |
| 40531 | 55_229 | E. cloacae | ST104 | CTX-M-9 |
| 40537 | 66_252_2 | E. cloacae | ST104 | CTX-M-9 |
| 40539 | 67_255_2 | E. cloacae | ST104 | CTX-M-9 |
| 41374 | 97_386 | E. cloacae | ND | CTX-M-1 |
| 41378 | 99_409_2 | E. cloacae | ND | CTX-M-9 |
| 41379 | 100_413_2 | E. cloacae | ND | CTX-M-9 |
| 41372 | 95_352_3 | E. cloacae | ND | CTX-M-9 |

**Figure S1.** PFGE profiles of *E. cloacae* isolates. Analysis was performed using the Bionumerics software, with an optimization and a tolerance set at 0.5%.

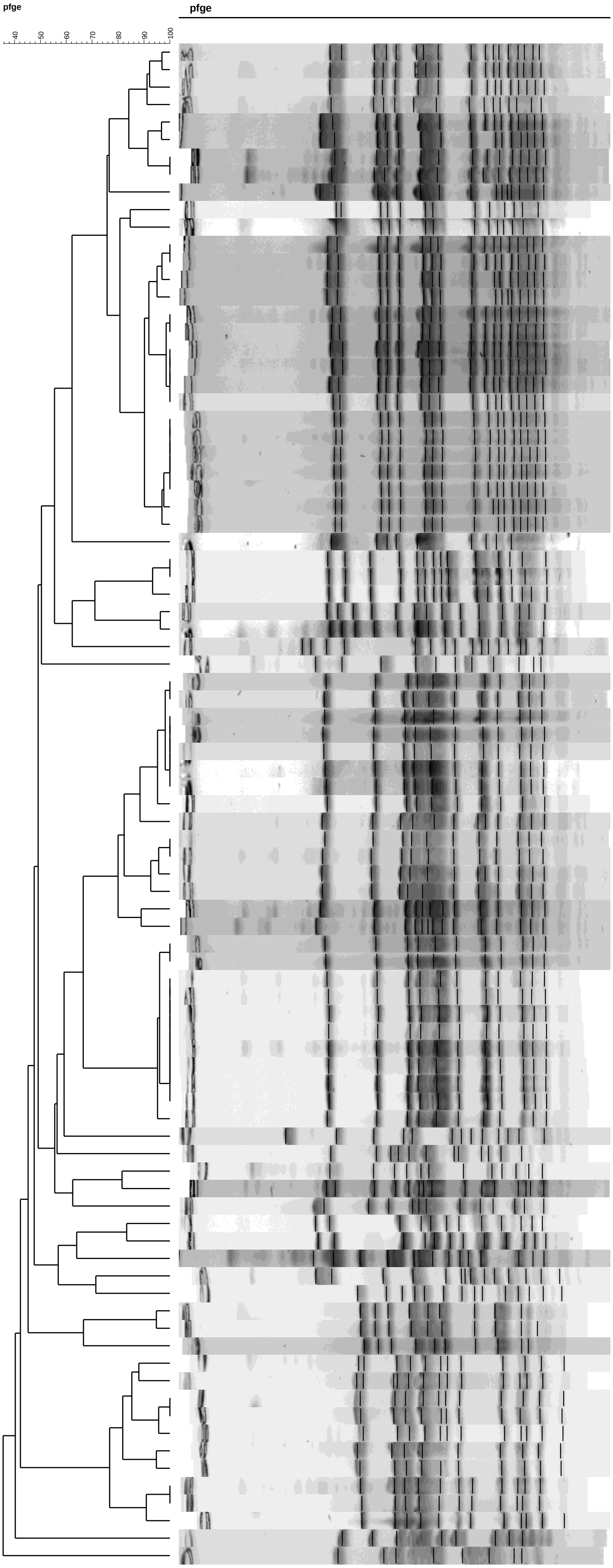


| Key | Bird number | Bacterial spec | MLST | CTX-M enzyme |
|---|---|---|---|---|
| 40480 | 69_258 | E. coli | ST746 | CTX-M-9 |
| 40483 | 73_270 | E. coli | ST746 | CTX-M-9 |
| 40496 | 86_311 | E. coli | ST746 | CTX-M-9 |
| 40482 | 72_268 | E. coli | ST746 | CTX-M-9 |
| 40475 | 65_251_2 | E. coli | ST746 | CTX-M-9 |
| 40476 | 66_252 | E. coli | ST746 | CTX-M-9 |
| 40444 | 32_175 | E. coli | ST746 | CTX-M-9 |
| 40456 | 41_195_2 | E. coli | ST746 | CTX-M-9 |
| 40478 | 68_257 | E. coli | ST746 | CTX-M-9 |
| 40428 | 25_131 | E. coli | ST746 | CTX-M-9 |
| 41381 | 95_352 | E. coli | ST746 | CTX-M-9 |
| 40468 | 56_230 | E. coli | ST746 | CTX-M-9 |
| 40469 | 58_232 | E. coli | ST746 | CTX-M-9 |
| 40470 | 60_240 | E. coli | ST746 | CTX-M-9 |
| 40474 | 65_251 | E. coli | ST746 | CTX-M-9 |
| 40464 | 50_220 | E. coli | ST746 | CTX-M-9 |
| 40466 | 52_222_2 | E. coli | ST746 | CTX-M-9 |
| 40459 | 44_206 | E. coli | ST746 | CTX-M-9 |
| 40460 | 45_209 | E. coli | ST746 | CTX-M-9 |
| 40467 | 53_225 | E. coli | ST746 | CTX-M-9 |
| 40488 | 79_297 | E. coli | ST746 | CTX-M-9 |
| 40439 | 31_165 | E. coli | ST746 | CTX-M-9 |
| 40445 | 33_176 | E. coli | ST746 | CTX-M-9 |
| 40449 | 37_184 | E. coli | ST746 | CTX-M-9 |
| 40451 | 38_186 | E. coli | ST746 | CTX-M-9 |
| 40457 | 42_196 | E. coli | ST746 | CTX-M-9 |
| 40454 | 40_190 | E. coli | ST746 | CTX-M-9 |
| 40453 | 39_189 | E. coli | ST746 | CTX-M-9 |
| 40501 | 93_334 | E. coli | ST746 | CTX-M-9 |
| 40414 | 13_96 | E. coli | ST40 | CTX-M-9 |
| 40415 | 14_97 | E. coli | ST40 | CTX-M-9 |
| 40418 | 16_102_2 | E. coli | ST40 | CTX-M-9 |
| 40495 | 85_310 | E. coli | ST4054 | CTX-M-9 |
| 40499 | 89_315 | E. coli | ST4054 | CTX-M-9 |
| 41380 | 76_276 | E. coli | ST1643 | CTX-M-9 |
| 40438 | 30_163 | E. coli | ST88 | CTX-M-1 |
| 40437 | 28_157 | E. coli | ST1246 | CTX-M-9 |
| 40485 | 75_273 | E. coli | ST1246 | CTX-M-9 |
| 40435 | 27_156 | E. coli | ST1246 | CTX-M-9 |
| 40448 | 36_183 | E. coli | ST1246 | CTX-M-9 |
| 40491 | 82_302 | E. coli | ST1246 | CTX-M-9 |
| 40498 | 88_313 | E. coli | ST1246 | CTX-M-9 |
| 40500 | 90_320 | E. coli | ST1246 | CTX-M-9 |
| 40424 | 23_125 | E. coli | ST1246 | CTX-M-9 |
| 40493 | 83_307 | E. coli | ST1246 | CTX-M-9 |
| 40486 | 77_282 | E. coli | ST1246 | CTX-M-9 |
| 40490 | 81_300 | E. coli | ST1246 | CTX-M-9 |
| 40494 | 84_309 | E. coli | ST1246 | CTX-M-9 |
| 40489 | 80_298 | E. coli | ST1246 | CTX-M-9 |
| 40465 | 52_222 | E. coli | ST1246 | CTX-M-9 |
| 40473 | 64_250 | E. coli | ST1246 | CTX-M-9 |
| 40450 | 37_184_2 | E. coli | ST1246 | CTX-M-9 |
| 40455 | 41_195 | E. coli | ST1246 | CTX-M-9 |
| 40407 | 6_56 | E. coli | ST1246 | CTX-M-9 |
| 40409 | 8_81 | E. coli | ST1246 | CTX-M-9 |
| 40410 | 9_84 | E. coli | ST1246 | CTX-M-9 |
| 40412 | 11_93 | E. coli | ST1246 | CTX-M-9 |
| 40413 | 12_95 | E. coli | ST1246 | CTX-M-9 |
| 40416 | 15_98 | E. coli | ST1246 | CTX-M-9 |
| 40422 | 20_116 | E. coli | ST1246 | CTX-M-9 |
| 40423 | 21_117 | E. coli | ST1246 | CTX-M-9 |
| 40419 | 17_111 | E. coli | ST1246 | CTX-M-9 |
| 40497 | 87_312 | E. coli | ST174 | CTX-M-9 |
| 41388 | 102_437 | E. coli | ST136 | CTX-M-1 |
| 40404 | 4_30 | E. coli | ST224 | CTX-M-1 |
| 40458 | 43_200 | E. coli | ST224 | CTX-M-1 |
| 41382 | 95_352_2 | E. coli | ST224 | CTX-M-1 |
| 40406 | 5_53 | E. coli | ST223 | CTX-M-9 |
| 40411 | 10_85 | E. coli | ST223 | CTX-M-9 |
| 40479 | 68_257_2 | E. coli | ST162 | CTX-M-9 |
| 40417 | 16_102 | E. coli | ST10 | CTX-M-1 |
| 40446 | 34_178 | E. coli | ST155 | CTX-M-1 |
| 40461 | 46_212 | E. coli | ST155 | CTX-M-1 |
| 40472 | 63_245 | E. coli | ST155 | CTX-M-1 |
| 40436 | 29_162 | E. coli | ST155 | CTX-M-1 |
| 40403 | 3_27 | E. coli | ST155 | CTX-M-1 |
| 40463 | 48_214 | E. coli | ST155 | CTX-M-1 |
| 40401 | 1_13 | E. coli | ST155 | CTX-M-1 |
| 40402 | 2_17 | E. coli | ST155 | CTX-M-1 |
| 40425 | 24_127 | E. coli | ST155 | CTX-M-1 |
| 40405 | 4_30_2 | E. coli | ST155 | CTX-M-1 |
| 40429 | 26_132 | E. coli | ST155 | CTX-M-1 |
| 41386 | 100_413 | E. coli | ST155 | CTX-M-1 |
| 41387 | 101_430 | E. coli | ST155 | CTX-M-1 |
| 40447 | 35_181 | E. coli | ST155 | CTX-M-1 |
| 40484 | 74_271 | E. coli | ND | CTX-M-9 |
| 40481 | 70_261 | E. coli | ND | CTX-M-9 |

**Figure S2.** PFGE profiles of *E. coli* isolates. Analysis was performed using the Bionumerics software, with an optimization and a tolerance set at 0.5%.

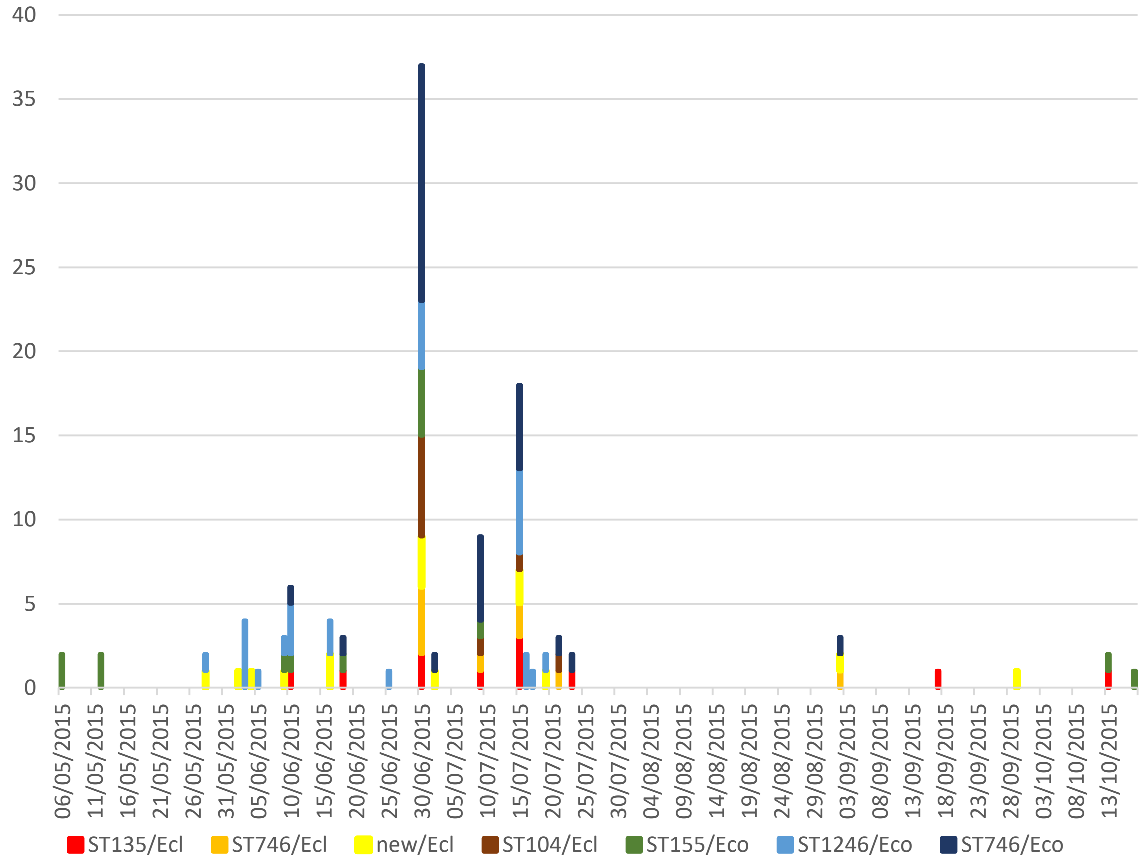


**Figure S3.** Epidemiological timeline of the principal *E. coli* and *E. cloacae* clones over time. Eco: *E. coli* and Ecl: *E. cloacae.*
